# Shoulder replacement: an epidemiological nationwide study from 2009 to 2019

**DOI:** 10.1186/s12891-022-05849-x

**Published:** 2022-09-30

**Authors:** Umile Giuseppe Longo, Rocco Papalia, Alessandro Castagna, Sergio De Salvatore, Enrico Guerra, Ilaria Piergentili, Vincenzo Denaro

**Affiliations:** 1 Research Unit of Orthopaedic and Trauma Surgery, Fondazione Policlinico Universitario Campus Bio-Medico, Via Alvaro del Portillo, 200, 00128 Roma, Italy; 2 Department of Medicine and Surgery, Research Unit of Orthopaedic and Trauma Surgery, Università Campus Bio-Medico Di Roma, Via Alvaro del Portillo, 21, 00128 Roma, Italy; 3 Shoulder Unit IRCCS Humanitas Institute, Milan, Italy; 4 Chirurgia Della Spalla E del Gomito, IRCCS Istituto Ortopedico Rizzoli, Bologna, Italy

**Keywords:** Shoulder replacement, Total Shoulder Replacement, Shoulder Hemiarthroplasty, Revision of shoulder joint replacement, Italy, Epidemiology, Costs

## Abstract

**Background:**

Shoulder replacement (SR) constitutes the gold standard treatment for severe shoulder diseases, including osteoarthritis, rheumatoid arthritis, complex fractures, avascular necrosis and rotator cuff arthropathy. Although several countries have national registries, there is a lack of epidemiological data on SR. Sharing national statistics and correlating those to other countries could be helpful to compare outcomes and costs internationally. This paper aims to evaluate the trend of hospitalizations for SR (both first implants and revisions of anatomical and reverse prosthesis) in Italy from 2009 to 2019, based on the National Hospital Discharge Reports (S.D.O) provided by the Italian National Health Service (INHS). Moreover, the economic impact on the healthcare system of SR and SR revisions was assessed, providing a statistical prediction for the next ten years.

**Methods:**

The data used in this paper were about patients who underwent Total Shoulder Replacement (TSR), Shoulder Hemiarthroplasty (SH) or Revision of shoulder joint replacement (RSR) from 2009 to 2019 in Italy. Information about patients was anonymous and included age, sex, days of hospitalization, procedures and diagnoses codes.

**Results:**

From 2009 to 2019, 73,046 TSR and SH were performed in adult Italian residents, with a cumulative incidence of 13.6 cases per 100,000 adult Italian residents. While, 2,129 revisions of shoulder replacement were performed, with a cumulative incidence of 0.4 cases per 100,000 residents. Overall, females represented the majority of the cases (72.4% of patients who underwent TSR or SH and 59.1% of patients who underwent RSR). From 2009 to 2019, has been assessed an overall cost of 625,638,990€ for TSR or SH procedures in Italy. While, an overall cost of 9,855,141€ for RSR procedures in Italy was calculated.

**Conclusions:**

The incidence of SR and RSR is expected to increase in the following years, constituting a burden for the healthcare systems. Overall, in Italy, the females represented the majority of patients. Further prospective studies on this topic in different countries can be con-ducted to make comparisons.

## Background

Shoulder replacement (SR) constitutes the gold standard treatment for severe shoulder diseases, including osteoarthritis, rheumatoid arthritis, complex fractures, avascular necrosis and rotator cuff arthropathy [1]. Several types of implants and designs have been proposed, providing adequate surgical solutions for different diseases [2–4].

The management of end-stage glenohumeral osteoarthritis is controversial; however, SR is currently the treatment of choice [5, 6]. However, also the psychological health status of the patient could influence the choice of treatment by the surgeon [7]. Each type of prosthesis, Total shoulder replacement (TSR) or Shoulder Hemiarthroplasty (SH), has advantages and drawbacks [8]. SH is technically easier and requires less operating time, reduced blood loss and lower costs [9, 10]. However, both operations may lead to severe postoperative complications [2, 11]. Recent studies have revealed that TSR provides better results than SH [12, 13]. This finding may justify the increase in TSR rate (anatomical and reverse TSR) and the decrease in SH. However, limited data are reported worldwide regarding clinical implant performances [14]. Several authors reported the lack of high-quality studies focused on the patients treated by SR (total shoulder arthroplasty, hemiarthroplasty, resurfacing hemiarthroplasty, total resurfacing and total reverse arthroplasty) [3, 15]. Moreover, also a Cochrane review by Craig et al. reported the lack of high-quality studies and the difficulty of establishing which implant or surgical technique is the most effective in different situations [16].

National registries constitute the most appropriate tool to monitor implant surveillance [17]. The Norwegian registries were created in 1994 and constitute the first shoulder arthroplasty register [14]. Other countries (New Zealand, United States, United Kingdome, Australia, Denmark, Sweden and Netherlands) started to report clinical and surgical records on a national register only in the last ten year [18, 19].

Epidemiological data from different national registries could help to provide information regarding SR performances and outcomes. Although several countries have national registries, there is a lack of epidemiological data on SR. Lübbeke and colleagues reported the lack of publication presenting the annual incidence and trends of SR in different countries [14]. For an international audience, national health statistics for SR could be interesting, as different surgical techniques and implant types are adopted between countries [15]. Sharing national statistics and correlating those to other countries could be helpful to compare outcomes and costs internationally [20]. According to Pace and colleagues [21], understanding the trend of SR could be helpful to establish an international consensus on the correct SR management, providing significant advantages for the related health service planning. Lastly, the economic impact of SR is progressively increasing, constituting a burden for the national healthcare systems [22]. To our knowledge, few studies assessed the economic incidence of SR on healthcare costs.

This paper aims to evaluate the trend of hospitalizations for SR (both first implants and revisions of anatomical and reverse prosthesis) in Italy from 2009 to 2019, based on the National Hospital Discharge Reports (S.D.O) provided by the Italian National Health Service (INHS). Sharing Italian national statistics could help to improve the global database on this topic. Based on the data, a statistical prediction of the incidence of SR hospitalizations was performed. Moreover, the economic impact on the healthcare system of SR and SR revisions was assessed, providing a statistical prediction for the next ten years.

## Methods

The INHS was founded in 1978, providing all citizens free access to medical care [23]. The INHS provided the database of this study. In the SDO records archive were collected data from both private and public hospitals. Official data on the healthcare services are collected by hospitals and local healthcare structures, entered into structured data files, and periodically sent to the Ministry of Health [24]. The data used in this paper were about patients who underwent Total Shoulder Replacement (TSR), Shoulder Hemiarthroplasty (SH) or Revision of shoulder joint replacement (RSR) from 2009 to 2019 in Italy. Information about patients was anonymous and included age, sex, days of hospitalization, procedures and diagnoses codes. Only patients with 81.80 (Total Shoulder Replacement), 81.81 (Partial Shoulder Replacement) or 81.97 (Revision Of Joint Replacement Of Upper Extremity) International Classification of Diseases, Ninth Revision, Clinical Modification (ICD-9-CM) procedures codes were included in the analyses. In the analysis was included only adult population. According to ISTAT [25, 26] the adult population was defined as patients with at last 15 years old. All methods were performed in accordance with the relevant guidelines and regulations.

### Statistical analysis

Descriptive statistical analyses were performed (means and standard deviation for continuous variables and frequencies and percentages for categorical variables). The incidence was calculated as the ratio between the number of cases and the size of the adult population (i.e., ≥ 15 years old), with reference to 100,000 adult Italian inhabitants (cases/population*100,000). Data about adult Italian residents were derived from the Italian National Institute of Statistics (ISTAT) database. The normality distribution was assessed using the Shapiro–Wilk test, and visually by histogram and kernel density plot. The Mann–Whitney U test was used to determine if there were statistically significant differences in age and length of hospitalization between males and females. The Kruskal–Wallis test was used to determine if there were statistically significant variations in the length of hospitalization across age groups. The Mann–Whitney U test with Bonferroni correction was used to analyze pairwise comparisons. The forecast model was performed with the AAA version of the Exponential Smoothing (ETS) algorithm without seasonality. All the statistical analyses were performed with the IBM SPSS Statistics for Windows, Version 26.0. (Armonk, NY: IBM Corp) and Statistical Analysis System (SAS) OnDemand for Academics.

## Results

### Total shoulder replacement and hemiarthroplasty

**Fig. 1:**
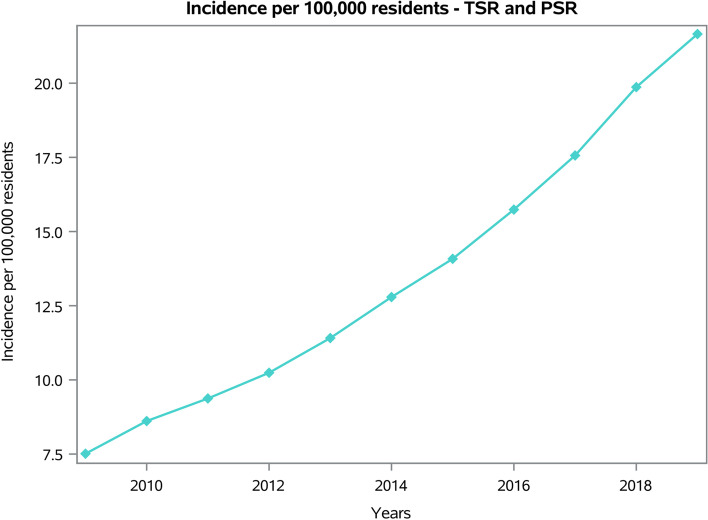
Incidence of TSR and SH procedures per 100,000 residents from 2009 to 2019

From 2009 to 2019, 73,046 shoulder replacements were performed in adult Italian residents, with a cumulative incidence of 13.6 cases per 100,000 adult Italian residents. The trend of the incidence was increasing, from a minimum of 7.5 in 2009 to a maximum of 21.7 cases per 100,000 residents in 2019 (Fig. 1).

**Fig. 2:**
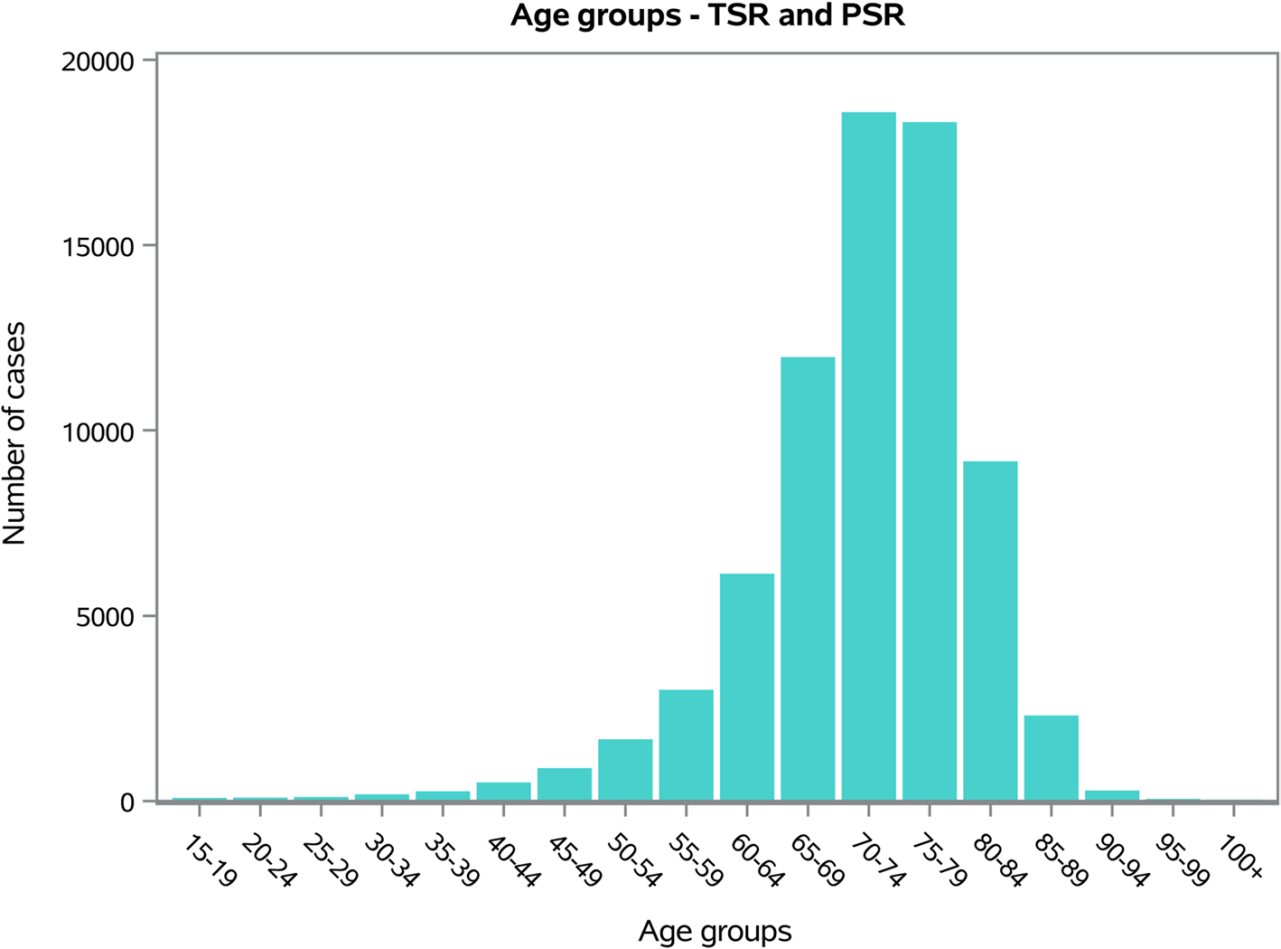
Frequencies of TSR and SH divided by age group

**Fig. 3:**
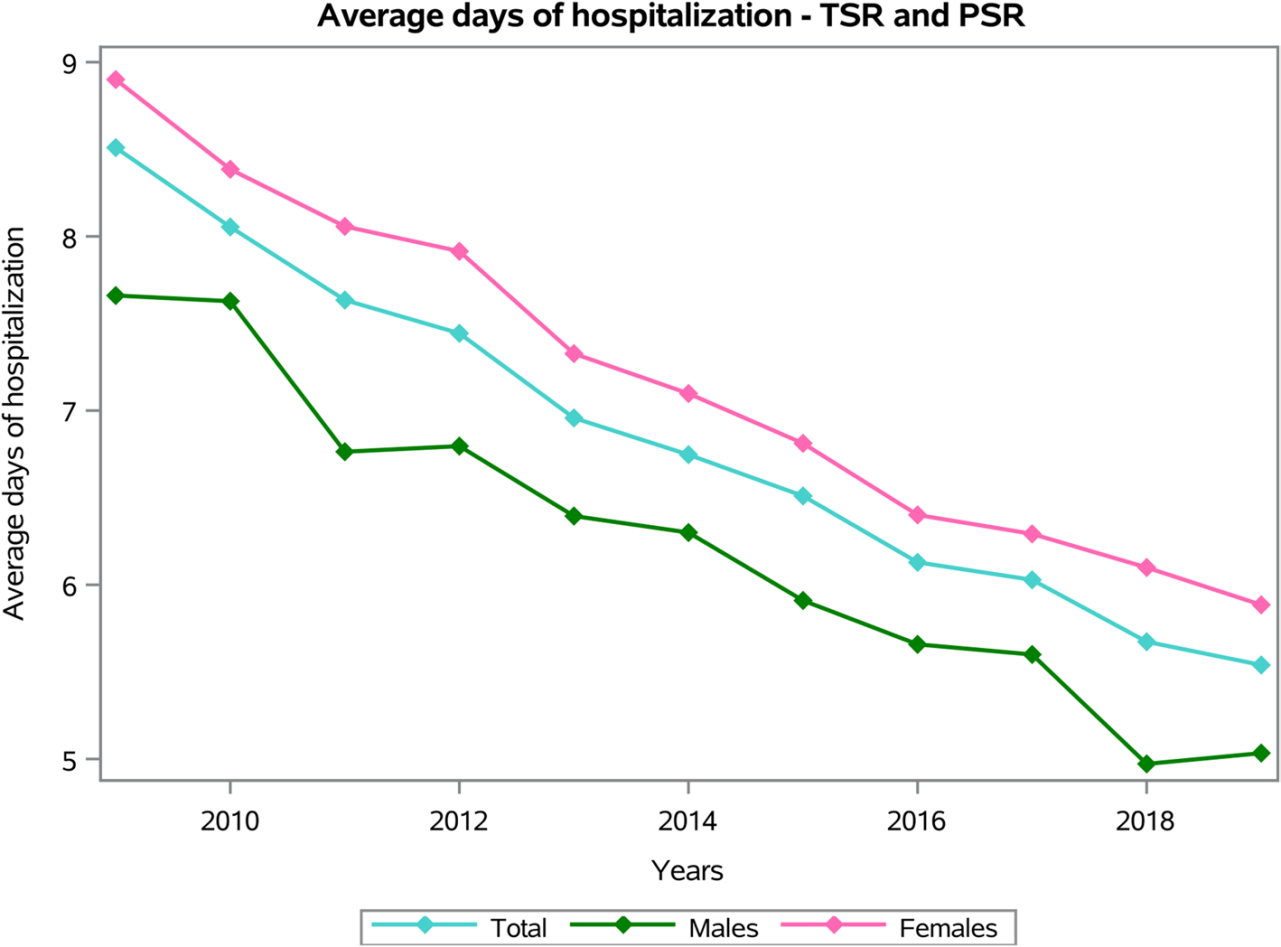
Average days of hospitalization by years of TSR and SH procedures

The males/females ratio was 0.4, and the females represented 72.4% of patients who underwent TSR or SH. Dividing by age groups, the most frequent were the 70–74 years old (25.4%) and the 75–79 years old (25%) (Fig. 2). The average age of patients was 71.54 ± 9.1 years (67.5 ± 11.2 years males and 72.8 ± 8.2 years females, *p* < 0.001). The overall mean days of hospitalization was 6.5 ± 5.4 days, with a decreasing trend from 8.5 ± 6.7 days in 2009 to 5.5 ± 4.4 days in 2019 (Fig. 3).

**Fig. 4:**
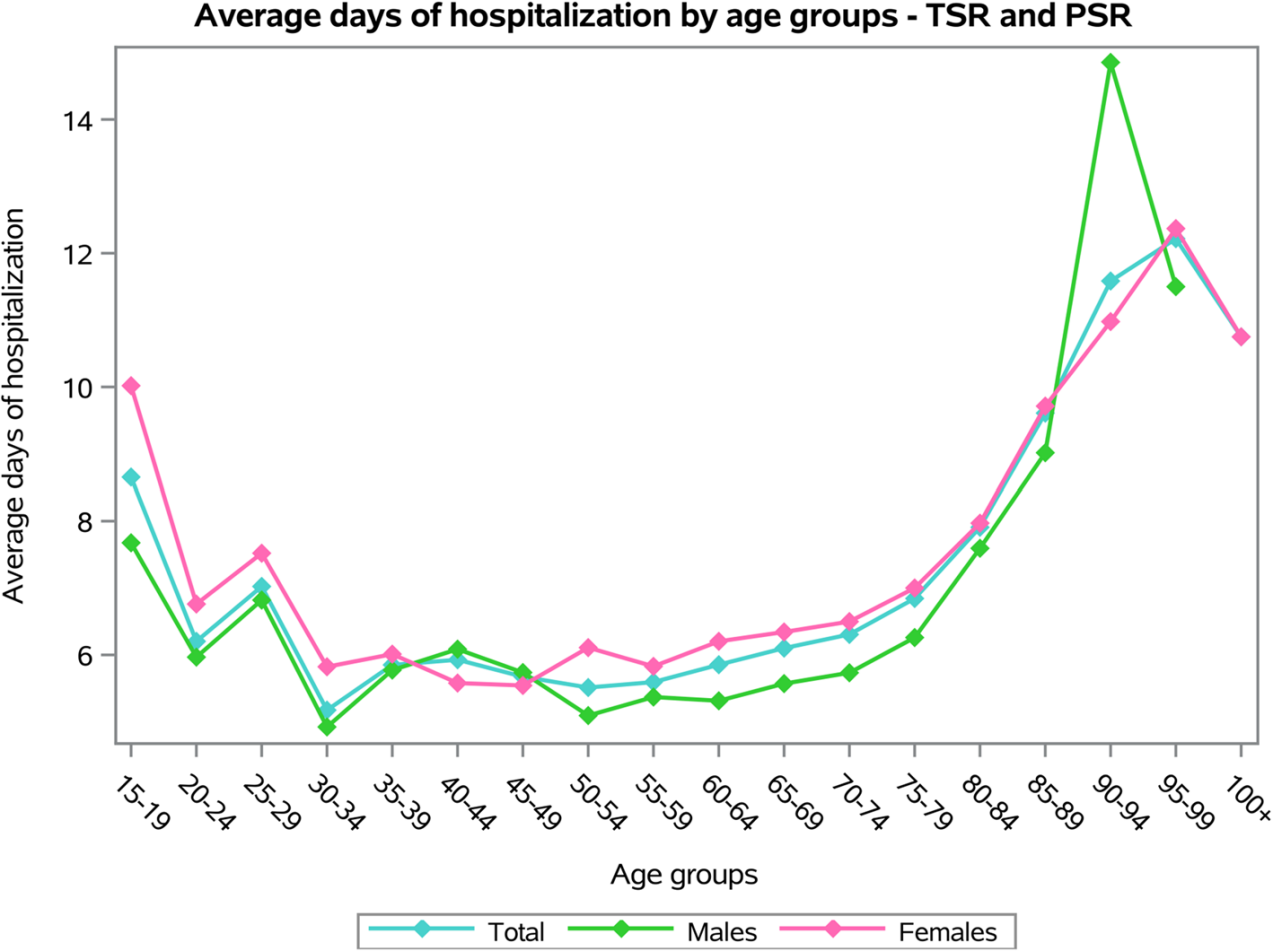
Average days of hospitalization by age groups of TSR and SH procedures

The females showed an average number of days of hospital stay higher than males (females 6.9 ± 5.7 days and males 5.9 ± 5.5 days, *p* < 0.001). Older patients presented the higher average days of hospital stay, both for males and females (*p* < 0.001) (Fig. 4).

The main primary diagnoses were “Osteoarthrosis, localized, primary, shoulder region" (715.11 ICD-9-CM code, 38%), “Closed fracture of unspecified part of upper end of humerus” (812.00 ICD-9-CM code, 13.6%), “Osteoarthrosis, localized, secondary, shoulder region” (715.21ICD-9-CM code, 8.1%), “Other closed fracture of upper end of humerus” (812.09 ICD-9-CM code, 7.4%) and “Complete rupture of rotator cuff” (727.61 ICD-9-CM code, 6.1%).

**Fig. 5:**
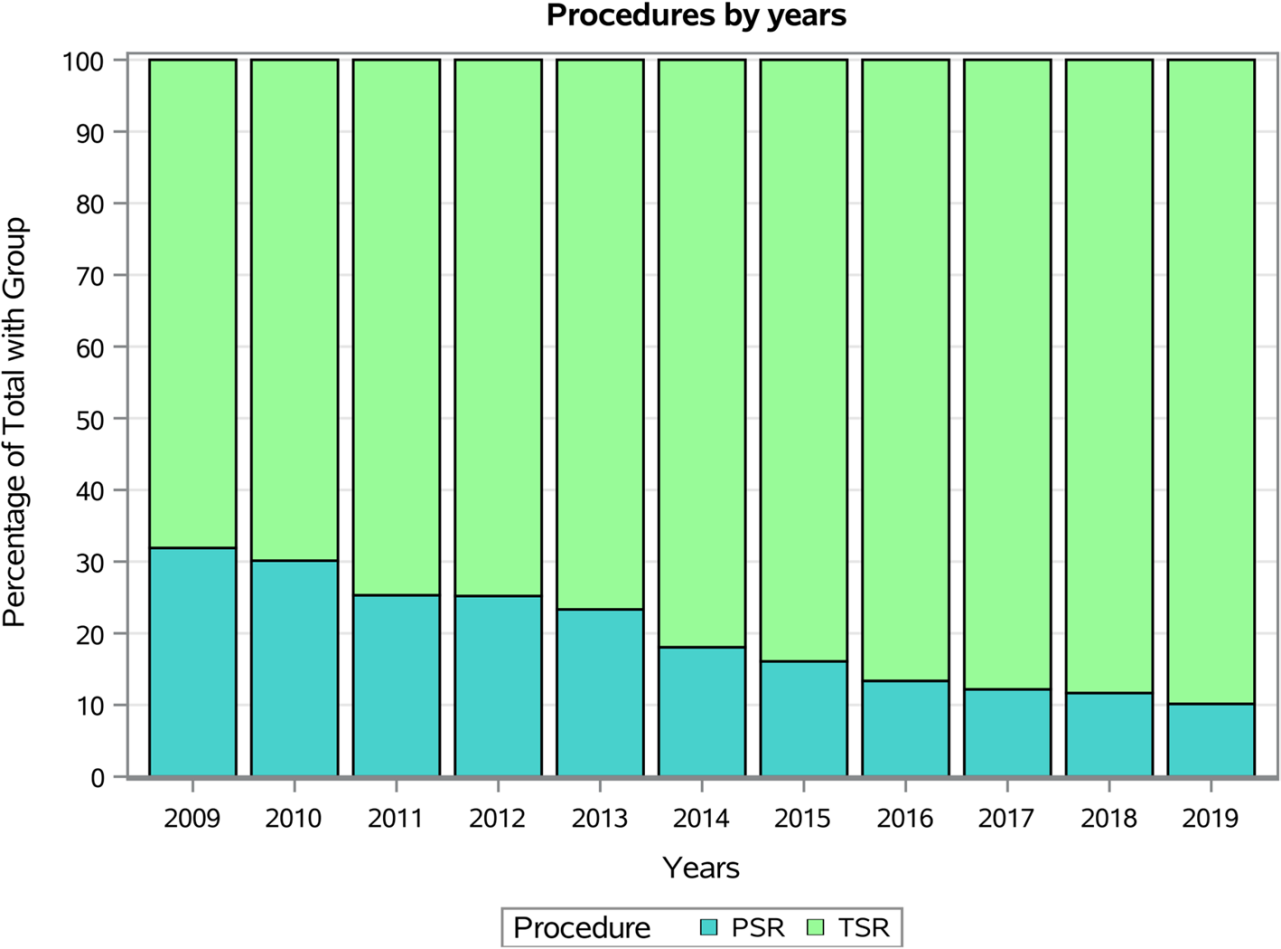
TSR and SH procedures by years

The most frequent procedure was TSR (ICD-9-CM code 81.80, 82.6%), in total and over the years (Fig. 5).

**Fig. 6:**
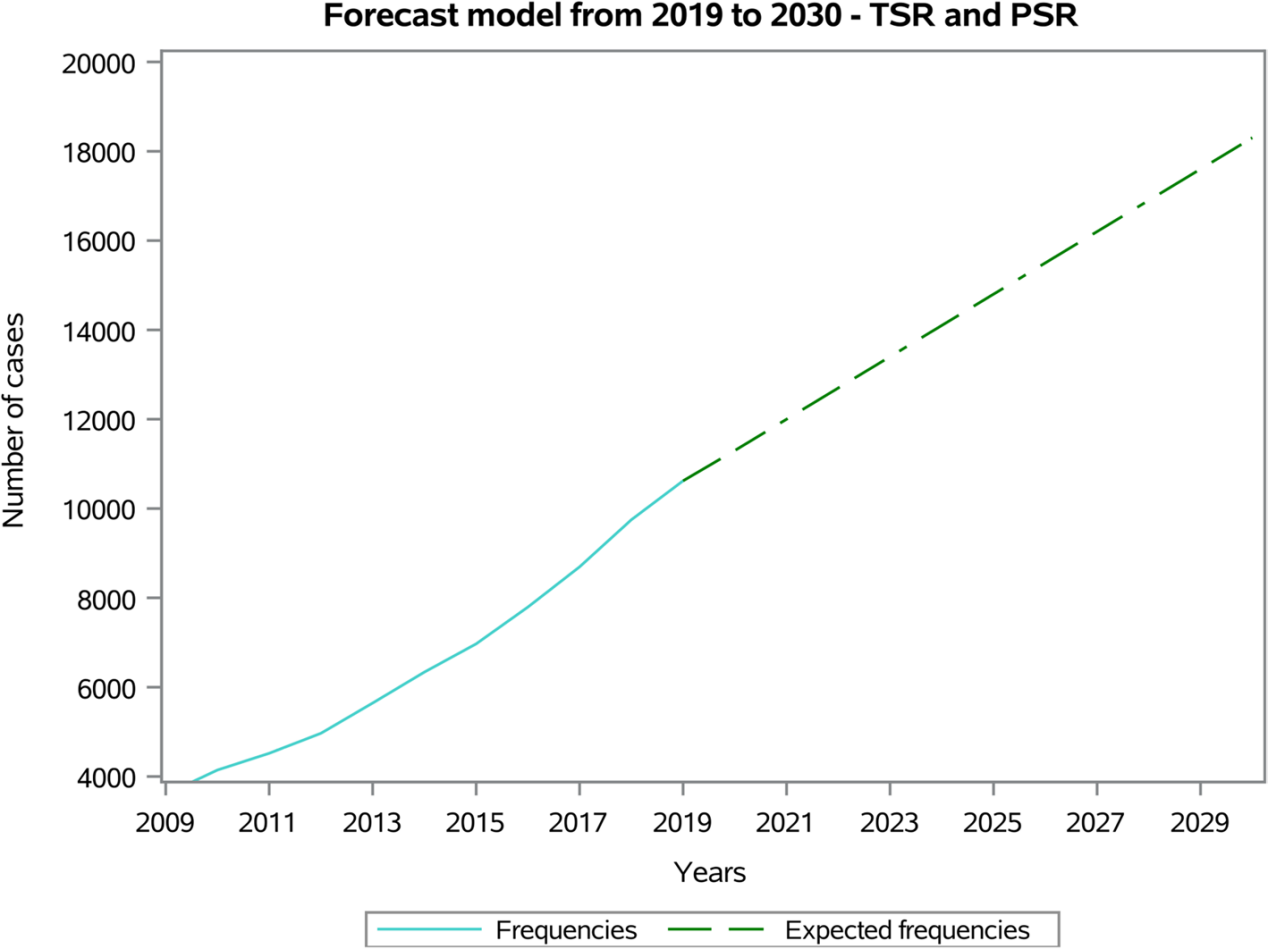
Forecast model from 2019 to 2030 of TSR and SH procedures

The forecast model predicted a growing trend from 10,621 procedures in 2019 to 183,02 procedures in 2030, with an increase of 72.3% in 2030 compared to 2019 (Fig. 6).

## Shoulder revisions

**Fig. 7:**
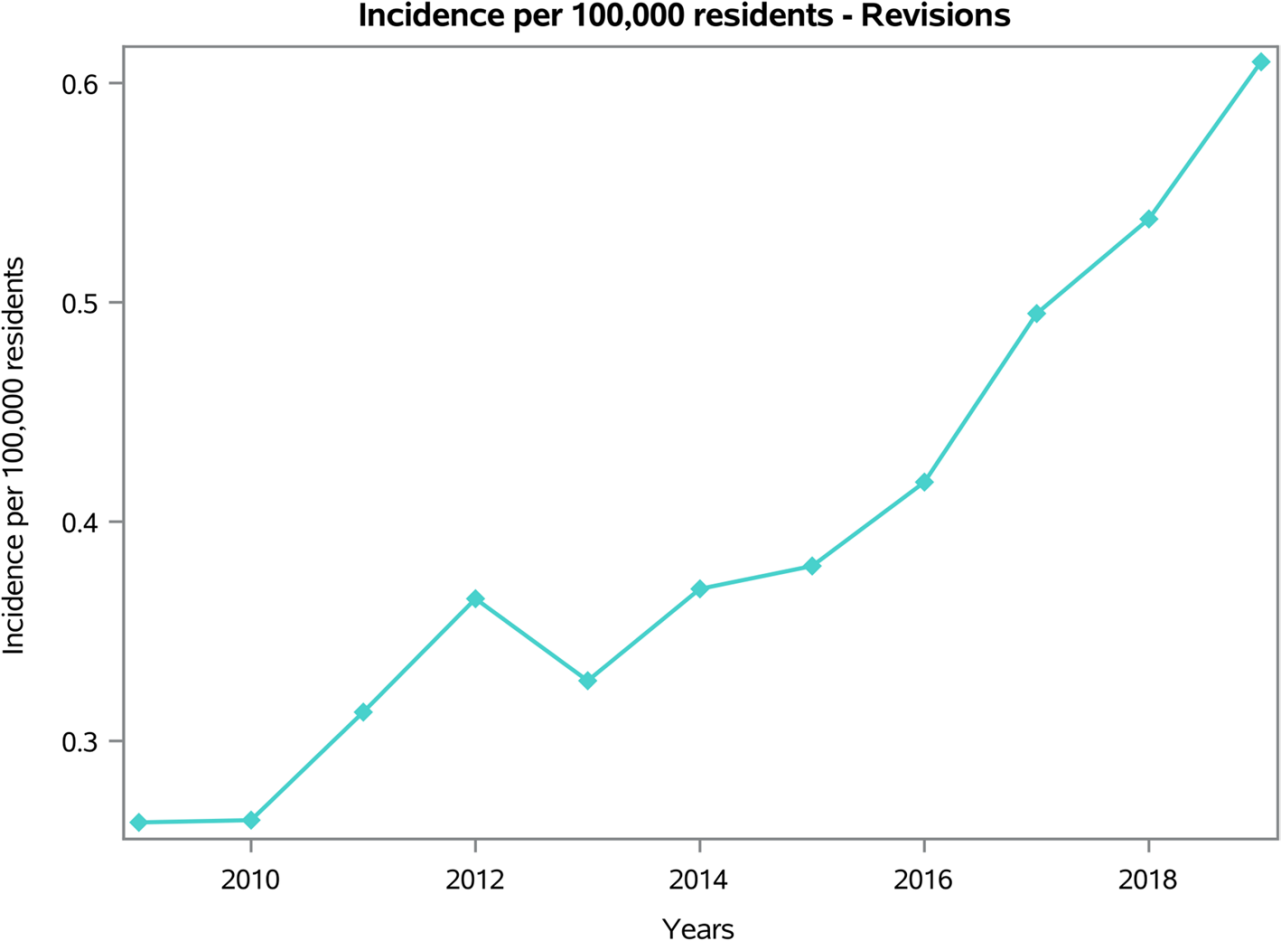
Incidence of RSR procedures per 100,000 residents from 2009 to 2019

From 2009 to 2019, 2,129 revisions of shoulder replacement were performed in adult Italian residents, with a cumulative incidence of 0.4 cases per 100,000 adult Italian residents. The trend of the incidence was increasing, from a minimum of 0.3 in 2009 to a maximum of 0.6 cases per 100,000 residents in 2019 (Fig. 7).

**Fig. 8:**
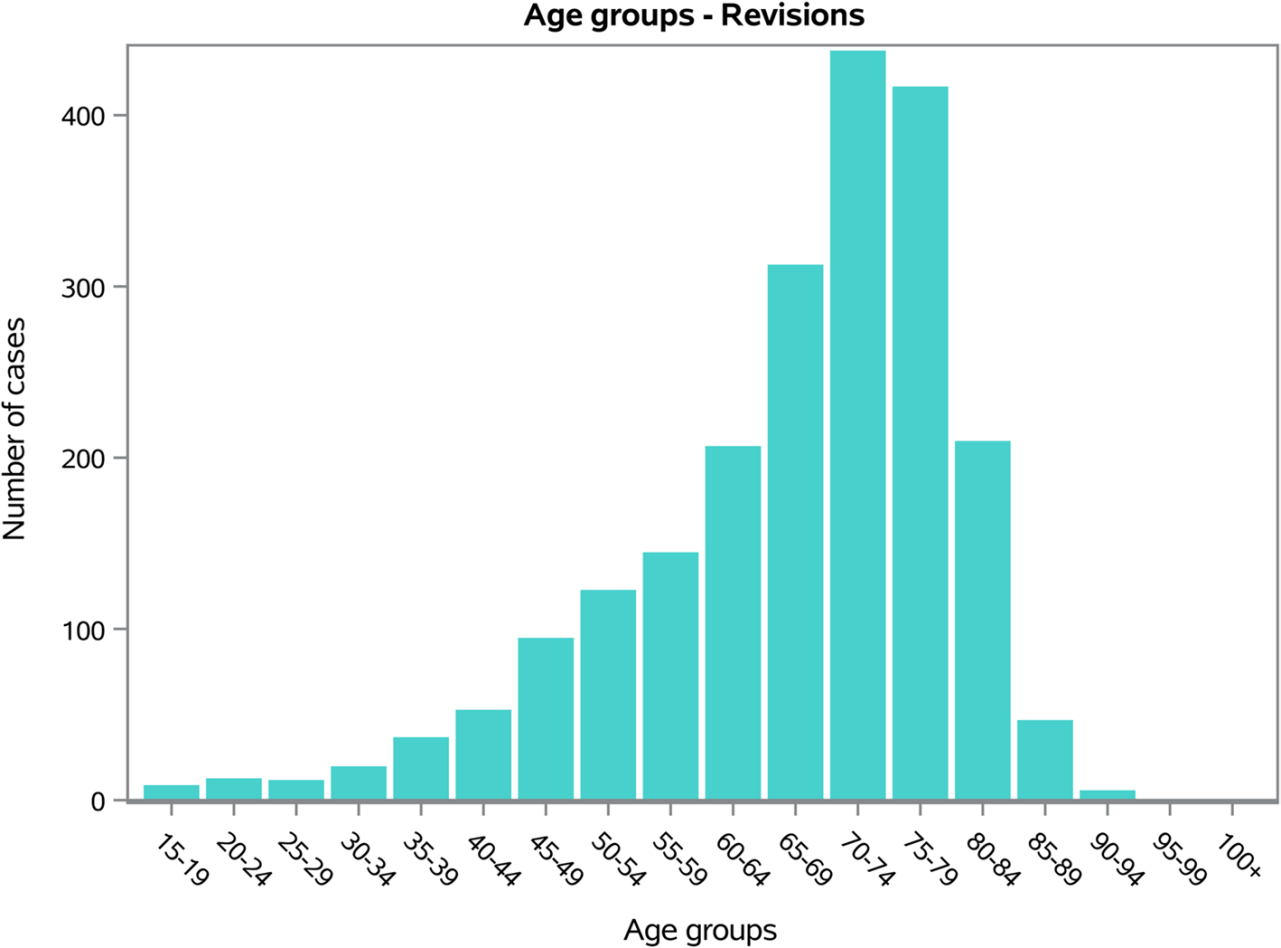
Frequencies of RSR divided by age group

**Fig. 9:**
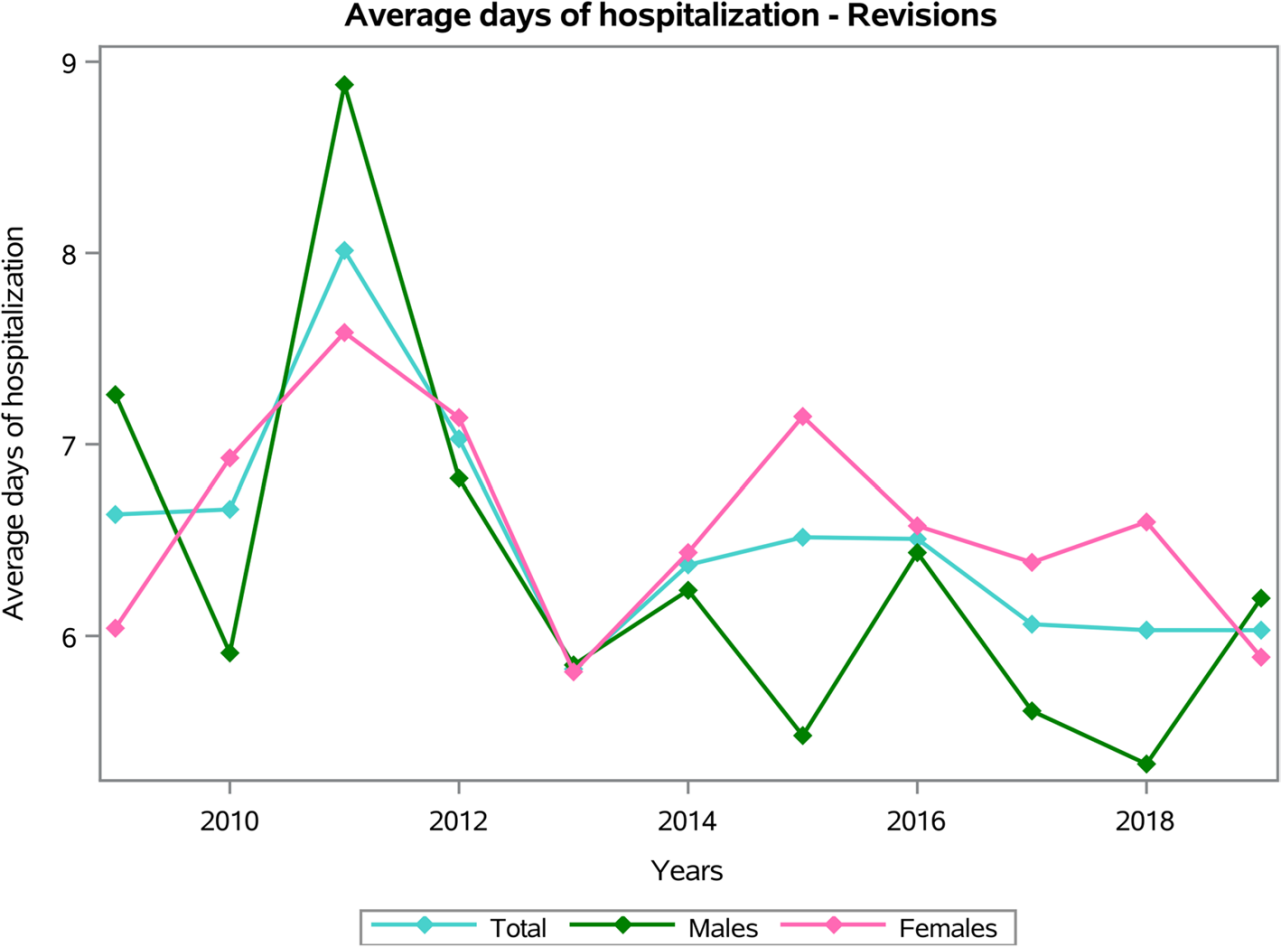
Average days of hospitalization by years of RSR procedures

The males/females ratio was 0.7, and the females represented 59.1% of patients who underwent shoulder arthroplasty revision. Dividing by age groups, the most frequent were the 70–74 years old (20.5%) and the 75–79 years old (19.5%) (Fig. 8). The average age of patients was 67 ± 12.9 years (63.3 ± 14.5 years males and 69.3 ± 11.6 years females, *p* < 0.001). The overall mean days of hospitalization was 6.4 ± 6.1 days, with a decreasing trend from 6.6 ± 5.1 days in 2009 to 6 ± 5.3 days in 2019 (Fig. 9).

**Fig. 10:**
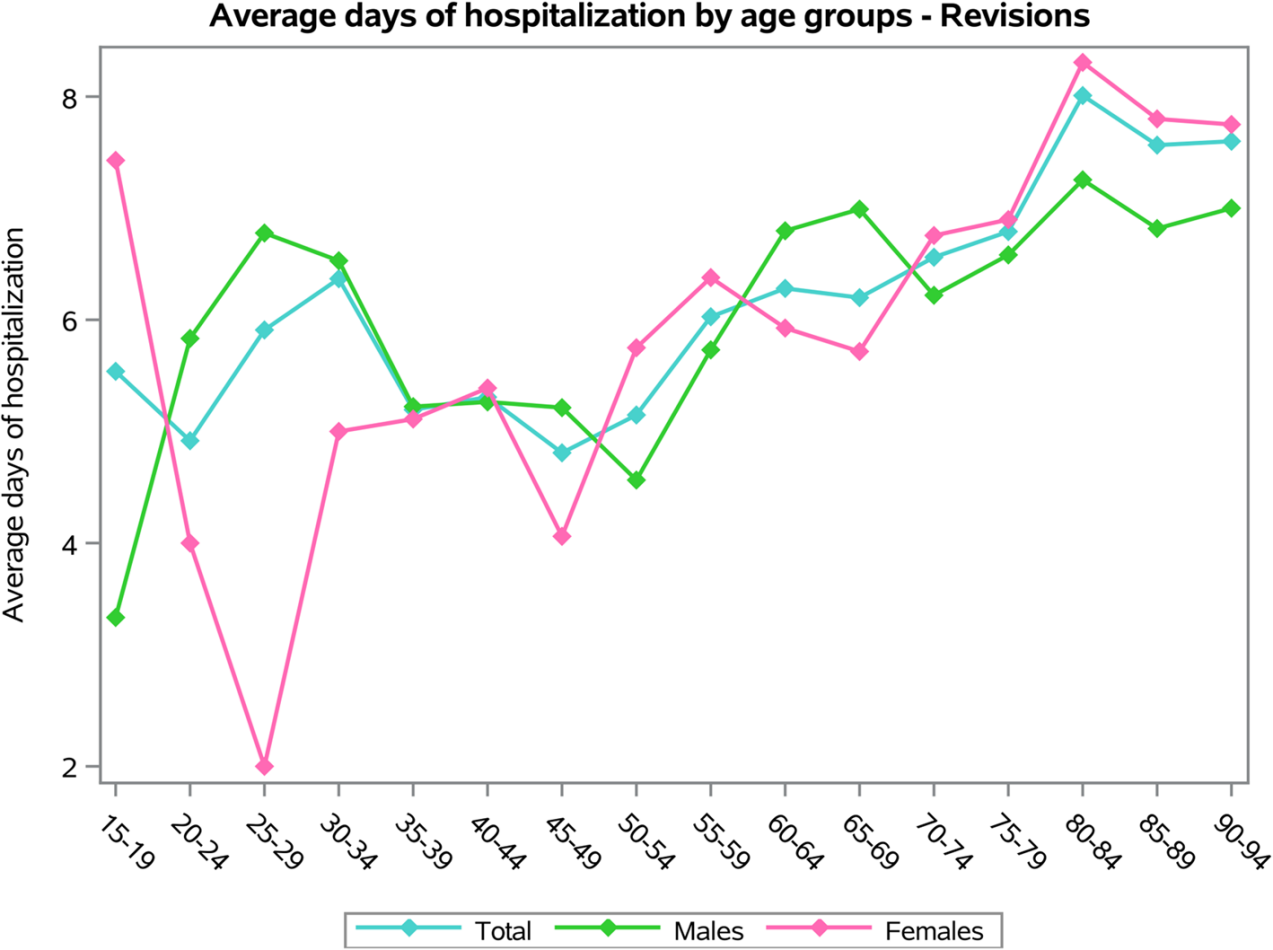
Average days of hospitalization by age groups of RSR procedures

No statistically significant differences between males and females in the average days of hospitalization (females 6.6 ± 6.2 days and males 6.2 ± 5.9 days, *p* = 0.104). Patients in the 80–84 age group presented the higher average days of hospital stay (Fig. 10). Older patients presented the higher average days of hospital stay, both for males and females (*p* < 0.001). Females between 15 and 19 and over 80 years old showed higher average days of hospital stay, while the higher average days of hospitalization in males were in age groups over 80 years old (Fig. 10).

The main primary diagnoses were “Dislocation of prosthetic joint " (996.42 ICD-9-CM code, 17.6%), “Unspecified mechanical complication of internal orthopedic device, implant, and graft” (996.40 ICD-9-CM code, 13.6%), “Mechanical loosening of prosthetic joint” (996.41 ICD-9-CM code, 10.8%), “Other mechanical complication of prosthetic joint implant” (996.47 ICD-9-CM code, 7.4%) and “Infection and inflammatory reaction due to internal joint prosthesis” (996.66 ICD-9-CM code, 6.1%).

**Fig. 11:**
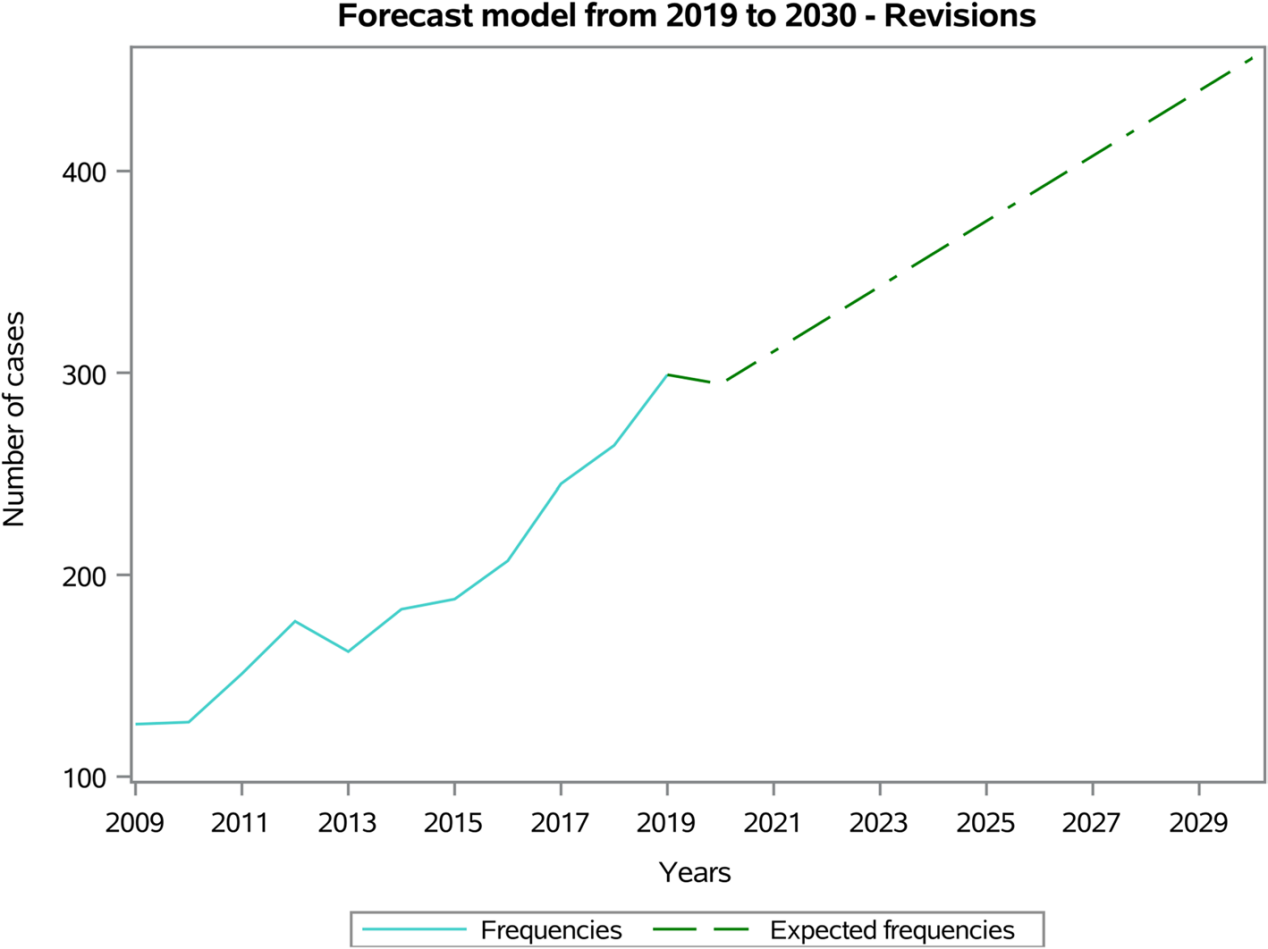
Forecast model from 2019 to 2030 of RSR procedures

The forecast model predicted a growing trend from 299 procedures in 2019 to 456 procedures in 2030, with an increase of 52.5% in 2030 compared to 2019 (Fig. 11).

### Economic impact

The actual mean Italian hospital reimbursement is 8,565€ for each TSR and SH hospital admission. In contrast, the Italian hospital reimbursement is 4,629€ for RSR hospital admission.

**Fig. 12:**
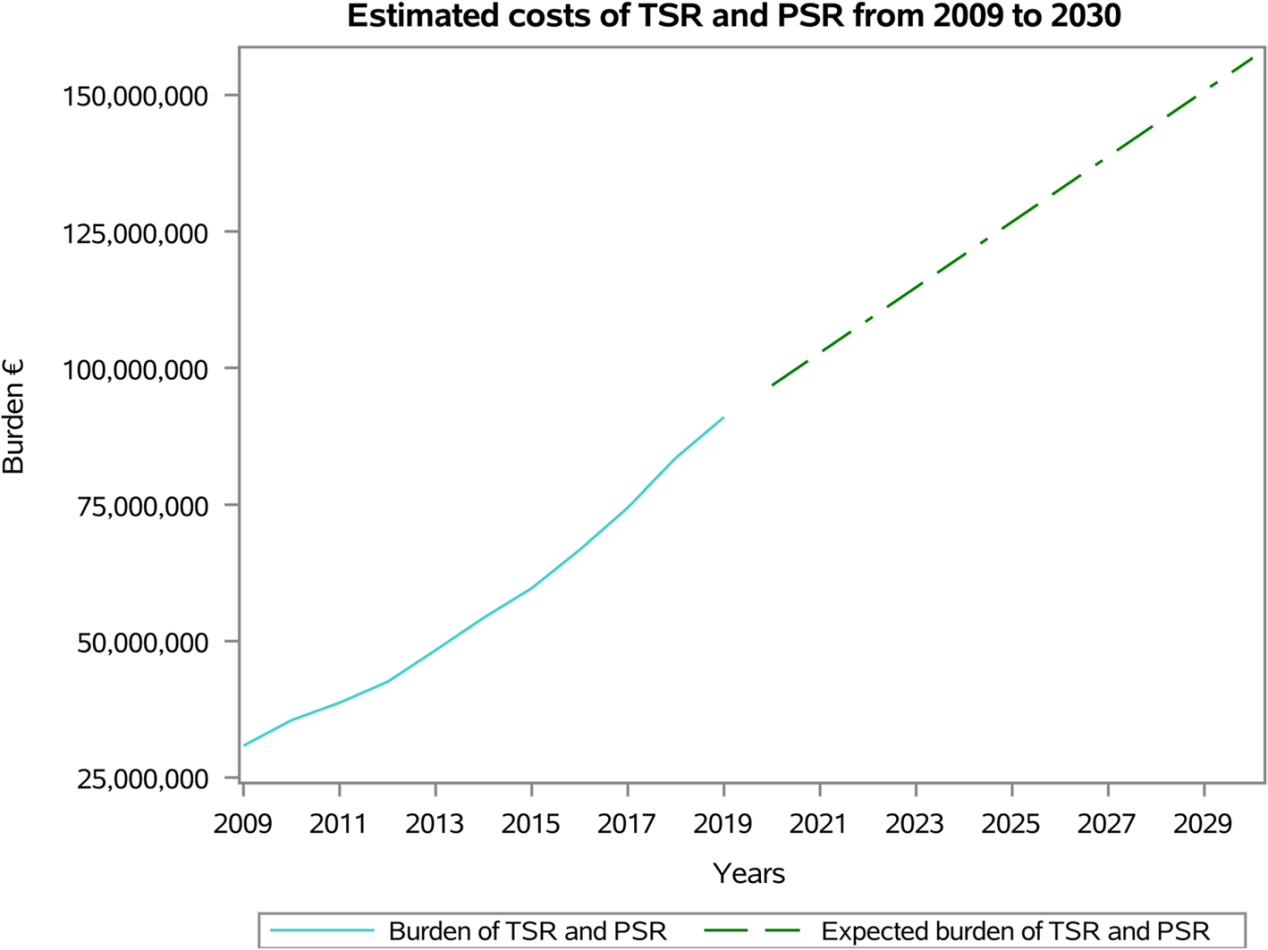
Estimated costs of TSR and SH procedures from 2009 to 2030

From 2009 to 2019, has been assessed an overall cost of 625,638,990€ (annual mean: 56,876,272€; ± 20,088,465€; range: from 30,842,565€ in 2009 to 90,968,865€ in 2019) for TSR or SH procedures in Italy (Fig. 12). Between 2020 and 2030, an overall cost of 1,394,479,987€ was estimated for TSR or SH procedures in Italy.

**Fig. 13:**
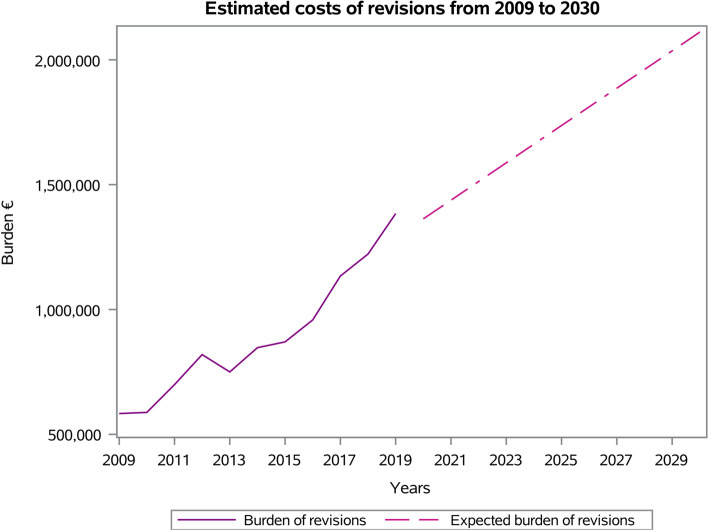
Estimated costs of RSR procedures from 2009 to 2030

An overall cost of 9,855,141€ (annual mean: 895,922€; ± 258,249€; range: from 583,254€ in 2009 to 1,384,071€ in 2019) for RSR procedures in Italy was calculated (Fig. 13). Between 2020 and 2030, an overall cost of 19,106,047€ was estimated for RSR procedures in Italy.

## Discussion

To our knowledge, this study is one of the largest studies ever published from a SR national registry. According to the forecast model, the results of this analysis reported a 3-fold increase of SR from 2009 to 2019 and a 72.3% increase in the next ten years.

This data is in accordance with the reports of European, American and Australian national registers [8, 27–30]. Day and colleagues [31] reported a progressive increase in SR in the United States population. Moreover, the national registry report of New Zealand showed an increasing number of hospitalization for SR [32]. The mean age of the patients included in the present analysis was similar to other international studies. In the United States, Jain and Adams [33, 34] reported that most SR were performed in the 65–79 years group. This data was also in accordance with other European and New Zealand studies [28, 29, 35]. According to the data of this study, 72.4% of patients who underwent TSR or SH were females. Rasmussen and colleagues [29] reported similar percentages, while Fevang et al. [28] described 85% of females in their population study. Instead, in the USA, according to Jain et al. [33], the number of female patients who underwent TSR progressively decreases during the years (from 66 to 57%). Similar to our findings, the studies by Adams, Jain and Kim reported osteoarthritis as the primary cause for SR [33, 34, 36].

TSR was the most commonly performed procedure in the present study as it was adopted in 82.6% of cases. Moreover, the overall incidence of TSR increased, while the rate of SH progressively decreased during the years. This data was in accordance with the study of Kim and colleagues [36]. However, the ICD-9-CM code cannot distinguish between TSA and reverse TSA (RTSA); therefore, the incidence of TSA recorded in the present study could be explained by the increased rate of RTSA performed worldwide [2]. Kim and colleagues [36] suggested that the overall increase of TSA recorded in the US population was due to the approval of RSTA by the United States Food and Drug Administration during the study period. However, the present study reported higher percentages of TSA performed compared to the Scandinavian registries. In Norway [28], SH represents the most common procedure performed and, in Denmark, TSR was used only in 3% of cases [18, 29]. However, the primary indication for SR in the Danish registry was “displaced humeral fracture” (54%) compared to only 13.6% in the present study.

The management of end-stage glenohumeral osteoarthritis is controversial; however, SR is currently the treatment of choice [5]. Each type of prosthesis, TSR or SH, has advantages and drawbacks [8]. SH is technically easier and requires less operating time, reduced blood loss and lower costs [9]. However, both operations may lead to severe postoperative complications [2, 11]. Recent studies have revealed that TSR provides better results than SH [12, 13]. This finding may justify the increase in TSR rate (anatomical and reverse TSR) and the decrease in SH.

The overall incidence of RSR of the present study was 0.4 cases per 100,000 adults.

Moreover, an increase in the trends of hospitalization for RSR during the years was reported. Due to ICD-9-CM limitations, it was not possible to identify the type of prosthesis revised (TSR, anatomical or reverse or SH); therefore, it was not possible to compare the results of the present study with other countries.

The economic burden of SR has almost doubled in the last ten years. The forecast model showed a progressive increase in the demand for SR and the consequent rise of the economic cost of this procedure. The rate of RSR will increase consequently, constituting a significant healthcare burden.

### Limitations

Administrative data from public and private hospitals were used in this research. For all procedures reported, the ICD-9 was adopted. Otherwise, different codes for the same surgical operation might be used with ICD-9. This coding heterogeneity could lead to an underestimation of our results. In addition, due to the ICD-9 limitation, it was impossible to discern between anatomical and reverse shoulder arthroplasties.

Moreover, a limit of the present study may be the lack of outcomes scores. However, Rasmussen and colleagues demonstrated no international consensus regarding using patient-reported outcome measures (PROMs) in national SR registries [18]. Polk and Fevang [28, 37] confirmed that the use of PROMs in SR registries could be challenging. Furthermore, patients did not receive a unique id number in the Italian healthcare system, as the hospitalization are anonymized. This means that patients who underwent more than one surgical procedure (particularly in RSR) were potentially counted twice or more. Additionally, the ICD-9 coding was performed by surgeons, and it results in individual inter-observer variations. Lastly, comparing the findings with other countries was difficult due to the differences in healthcare systems.

## Conclusions

The results of these studies highlighted that the indications for elective SR might differ worldwide. Moreover, the incidence of SR and RSR is expected to increase in the following years, constituting a burden for the healthcare systems. Therefore, epidemiological studies may help provide the necessary data for establishing international guidelines concerning the indication and outcome measure for SR.

## Data Availability

The datasets used and/or analyzed during the current study are not publicly available due on our policy statement of sharing clinical data only on request but are available from the corresponding author on reasonable request. The access to the database is on request. All data were obtained by the Direzione Generale della Programmazione Sanitaria—Banca Dati SDO of the Italian Ministry of Health.

## Abbreviations

ETS: Exponential Smoothing algorithm
ICD-9-CM: International Classification of Diseases, Ninth Revision, Clinical Modification
INHS: Italian National Health Service
ISTAT: Italian National Institute of Statistics
S.D.O: National Hospital Discharge Reports
PROMs: Patient-reported outcome measures
RSR: Revision of shoulder joint replacement
SH: Shoulder Hemiarthroplasty
SR: Shoulder replacement
TSR: Total Shoulder Replacement
